# Ivabradine improves survival and attenuates cardiac remodeling in isoproterenol‐induced myocardial injury

**DOI:** 10.1111/fcp.12620

**Published:** 2020-11-07

**Authors:** Fedor Simko, Tomas Baka, Kristina Repova, Silvia Aziriova, Kristina Krajcirovicova, Ludovit Paulis, Michaela Adamcova

**Affiliations:** ^1^ Institute of Pathophysiology Faculty of Medicine Comenius University Bratislava 81108 Slovak Republic; ^2^ 3rd Department of Internal Medicine Faculty of Medicine Comenius University Bratislava 83305 Slovak Republic; ^3^ Institute of Experimental Endocrinology Biomedical Research Center Slovak Academy of Sciences Bratislava 84505 Slovak Republic; ^4^ Department of Physiology School of Medicine Charles University Hradec Kralove 50003 Czech Republic

**Keywords:** heart hypertrophy, isoproterenol, ivabradine, mortality, remodeling, survival

## Abstract

This study investigated whether ivabradine, a selective I_f_ current inhibitor reducing heart rate (HR), is able to improve survival and prevent left ventricular (LV) remodeling in isoproterenol‐induced heart damage. Wistar rats were treated for 6 weeks: controls (*n* = 10), ivabradine (10 mg/kg/day orally; *n* = 10), isoproterenol (5 mg/kg/day intraperitoneally; *n* = 40), and isoproterenol plus ivabradine (*n* = 40). Isoproterenol increased mortality, induced hypertrophy of both ventricles and LV fibrotic rebuilding, and reduced systolic blood pressure (SBP). Ivabradine significantly increased survival rate (by 120%) and prolonged average survival time (by 20%). Furthermore, ivabradine reduced LV weight and hydroxyproline content in soluble and insoluble collagen fraction, reduced HR and attenuated SBP decline. We conclude that ivabradine improved survival in isoproterenol‐damaged hearts.

Although several effective approaches to heart failure (HF) treatment have been introduced during the recent decades, the high residual mortality stimulates the continuous search for novel therapeutic interventions [1]. Elevated resting heart rate (HR) in HF deteriorates the prognosis. Beta‐blockade is considered a cornerstone of HF treatment. Yet, beta‐blockers reduce contractility, limiting the achievement of recommended doses and sufficient HR decline [2]. The unique bradycardic drug, ivabradine, selectively inhibits the pacemaker I_f_ current in the sinoatrial node, thus slowing the diastolic depolarization resulting in HR reduction without a negative inotropic effect. In the SHIFT trial with systolic HF and HR above 70 bpm, ivabradine reduced the composite endpoint of mortality and hospitalization for HF [2]. Nonetheless, in the BEAUTIFUL study with systolic HF patients and HR above 70 bpm suffering from ischemic heart disease, the mortality outcomes were neutral. The missing effect of ivabradine on hard endpoints in the BEAUTIFUL study was presumably determined by the ischemic etiology of HF, since hypothetically, the loss of cardiomyocytes via ischemic death limits the volume of dysfunctional yet subsisting myocardium, which could be protected by therapeutic interventions [3]. In clinical trials, however, ivabradine was always complementary to a well‐established standard HF treatment, and there is a shortage of data on the potential benefit of ivabradine alone (i.e., without the established therapy) in myocardial ischemic damage.

The aim of our study was to show whether ivabradine is able to improve survival and interfere with heart remodeling in isoproterenol‐induced cardiac damage.

Four groups of 3‐month‐old male Wistar rats were treated for six weeks as follows: controls (C; 10 animals), rats treated with ivabradine (Iva; 10 animals, 10 mg/kg/day orally; Servier, France), isoproterenol (Iso; 40 rats, 5 mg/kg/day intraperitoneally; Sigma‐Aldrich, Germany), and isoproterenol plus ivabradine (Iso + Iva; 40 rats treated in corresponding doses). Both C and Iva groups were injected saline vehicle intraperitoneally as control to isoproterenol administration. The Iva and Iso + Iva groups were also administered ivabradine (10 mg/kg/day orally) as a pretreatment one week before the experiment began. Rats were maintained conforming to the Guide for the Care and Use of Laboratory Animals by the US National Institutes of Health. The protocol was approved by the ethical committee of the Institute of Pathophysiology, Faculty of Medicine, Comenius University, Bratislava, Slovak Republic (approval number: 1306/14‐221).

Systolic blood pressure (SBP) and heart rate (HR) were measured two weeks before the experiment started and after each week of treatment by noninvasive tail‐cuff plethysmography (Hugo Sachs Elektronic, Germany). After 6 weeks of the experiment, the rats were euthanized by isoflurane inhalation and their body weight (BW) and heart weight, left and right ventricle weights (LVW and RVW), and relative ventricular weights (LVW/BW and RVW/BW ratio) were determined. LV samples were frozen at −80°C and used for hydroxyproline measurement. As described previously [4], the soluble collagenous proteins were extracted with 0.5 mol/L CH3COOH‐pepsin buffer and the remaining insoluble collagenous proteins with 1.1 mol/L NaOH. Hydroxyproline was estimated using spectrophotometry at 550 nm. The results are expressed as mean ± SEM. One‐way, two‐tailed analysis of variance (anova) followed by multiple comparisons test with Bonferroni correction was used for statistical analysis. Kaplan–Meier method and log‐rank test were used for survival analysis. Differences were considered significant if the *P*‐value < 0.05. The statistical analysis was conducted using GraphPad Prism 8 (La Jolla, USA).

The survival rate of both control and ivabradine groups was 100%. Isoproterenol decreased the survival rate (10 of the 40 animals survived for 42 days), and the average survival time was 25.9 days. Ivabradine cotreatment increased (*P* < 0.05) the survival rate (by 120%; 22 of the 40 animals survived for 42 days) and the average survival time to 31 days (by 20%) (*Figure* *1a* and *b*).

**Figure 1:**
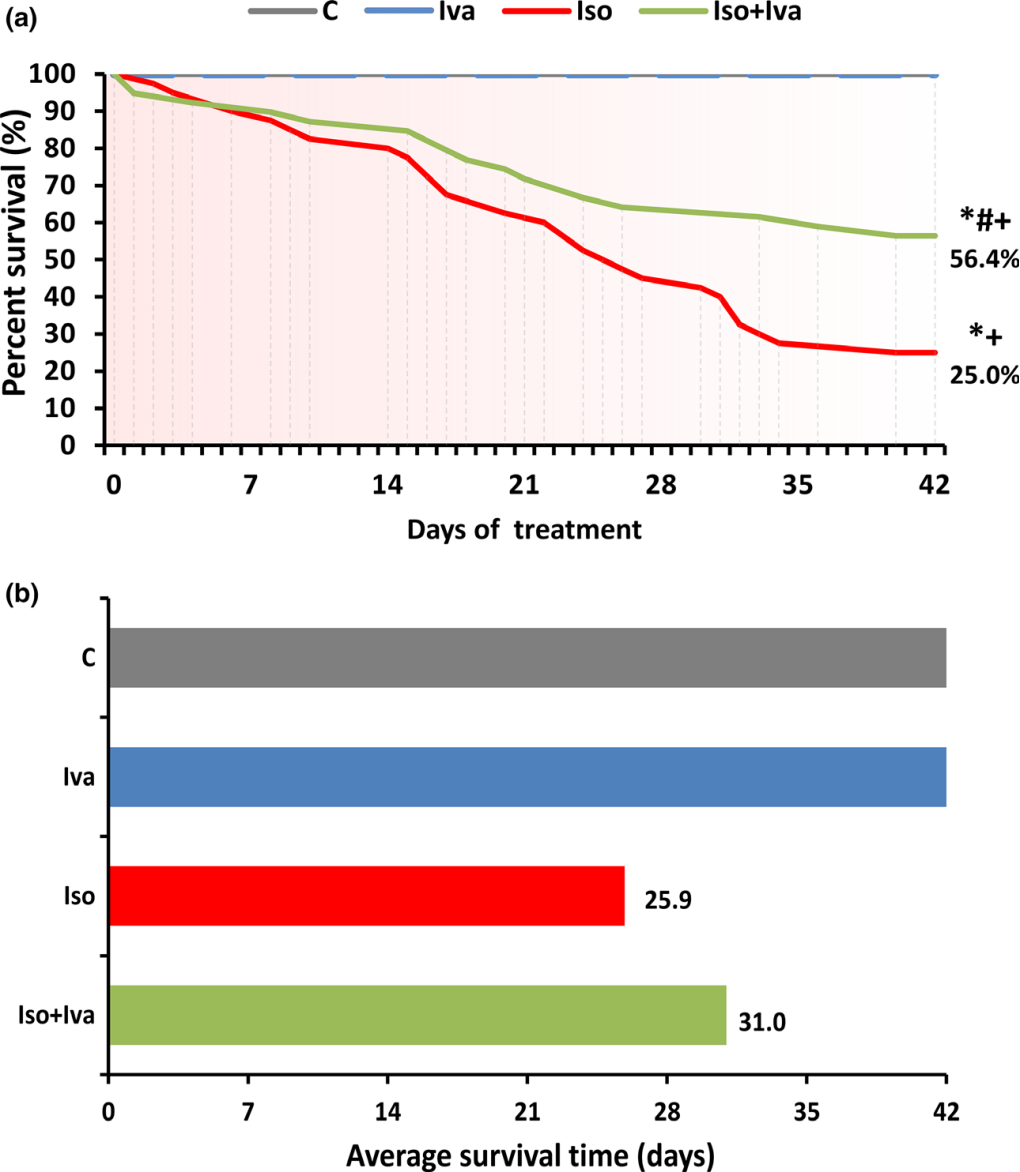
The effect of ivabradine (Iso + Iva) on survival in % (a) and average survival time (b) in isoproterenol‐induced myocardial injury (Iso). C, control; Iva, ivabradine; Iso, isoproterenol; *P < 0.05 vs. C; #*P* < 0.05 vs. Iso; +*P* < 0.05 vs. Iva.

The SBP was 127 ± 1 mmHg in the control group and 6‐week isoproterenol administration decreased it by 31% (*P* < 0.05). Ivabradine cotreatment increased SBP by 16% (*P* < 0.05) (*Figure* *2a*). HR was 390 ± 1 bpm in controls and was unchanged by isoproterenol. Ivabradine reduced HR in the control and isoproterenol groups (by 34% and 18%, respectively; *P* < 0.05) (*Figure* *2b*). The LVW/BW was 1.29 ± 0.05 mg/g in the control group and 6‐week isoproterenol administration increased it by 87% (*P* < 0.05); ivabradine cotreatment reduced LVW/BW by 21% (*P* < 0.05) (*Figure* *2c*). The RVW/BW was 0.56 ± 0.02 mg/g in the control group and 6‐week isoproterenol administration increased it by 62% (*P* < 0.05); ivabradine cotreatment left RVW/BW unchanged (*Figure* *2d*).

**Figure 2:**
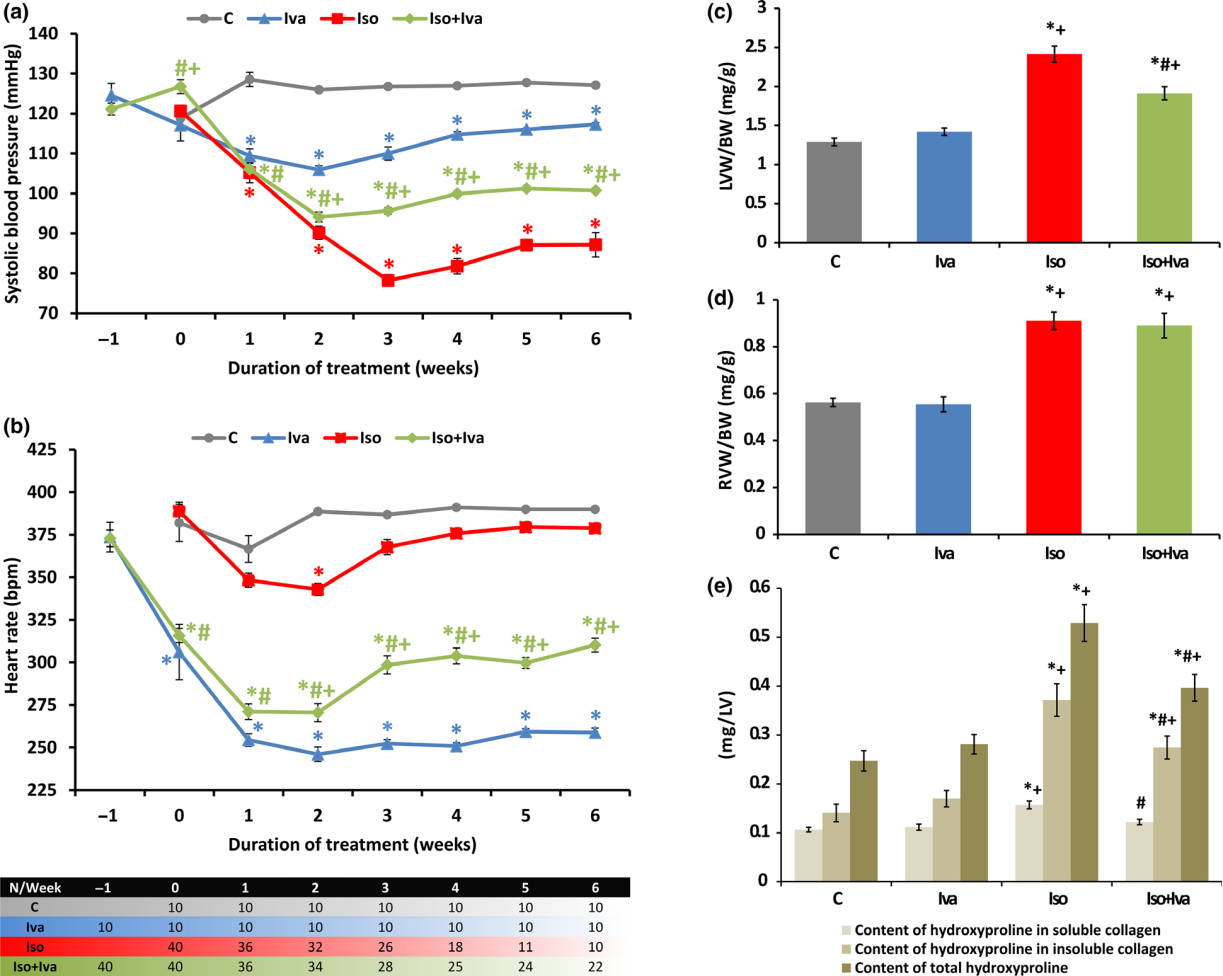
The effect of ivabradine (Iso + Iva) on systolic blood pressure (a), heart rate (b), relative left ventricular weight (LVW/BW) (c), relative right ventricular weight (RVW/BW) (d), and hydroxyproline content in soluble and insoluble collagenous proteins and on total hydroxyproline content in LV (e) in isoproterenol‐induced myocardial injury (Iso). C, control; Iva, ivabradine; Iso, isoproterenol; For a,b: *N* per week of treatment depicted in table; For c,d,e: *N* = 10 (in C, Iva, and Iso) or 22 (in Iso + Iva); *P < 0.05 vs. C; #*P* < 0.05 vs. Iso; +*P* < 0.05 vs. Iva.

Isoproterenol increased (*P* < 0.05) hydroxyproline content in both soluble and insoluble collagenous proteins vs. controls (0.16 ± 0.01 mg/LV vs. 0.11 ± 0.01 mg/LV and 0.37 ± 0.03 mg/LV vs. 0.14 ± 0.02 mg/LV, respectively). The total hydroxyproline content was also higher (114%; *P* < 0.05) in the isoproterenol group vs. controls. Cotreatment with ivabradine reduced (*P* < 0.05) the hydroxyproline content in both soluble and insoluble collagenous proteins by 22% and 26%, respectively, and the total hydroxyproline content by 25% (*Figure* *2e*).

The pathogenesis of the heart damage by isoproterenol, a synthetic catecholamine stimulating beta‐adrenergic receptors, involves overstimulation of the heart contractility, energy depletion, calcium overload, and oxidative stress. These mechanisms underlie myocardial injury manifested as cardiac ischemia, multiple necroses, LV dysfunction, and HF [1, 5, 6]. Here, in line with our previous experiment [1], the isoproterenol‐induced LV remodeling was characterized by the hypertrophy of both ventricles and LV fibrotic rebuilding. Ivabradine reduced the relative weight of the hypertrophied LV along with the diminution of the hydroxyproline content in all three collagen fractions. Besides HR reduction, ivabradine attenuated SBP decline. Our results are in agreement with previous findings in angiotensin II‐induced hypertension, where ivabradine improved LV function and reduced LV hypertrophy and fibrosis [7], and in cholesterol‐fed rabbits, where ivabradine reduced cardiac fibrosis and improved LV diastolic function [8]. Similarly, HR reduction by ivabradine for 36 months reduced LV mass index in heart transplant recipients [9]. In rats with postmyocardial infarction HF, ivabradine reduced interstitial fibrosis and improved both LV ejection fraction and end‐diastolic pressure [10]. Nevertheless, in L‐NAME‐induced hypertension, ivabradine improved LV function without attenuating LV collagen levels or hypertrophy [4]; and in thyroid hormone‐induced heart enlargement, ivabradine improved LV function without affecting ventricular remodeling [11]. Ivabradine’s effect on LV rebuilding appears to depend on the type and severity of heart alteration.

Several mechanisms could underlie the increased survival rate and average survival time by ivabradine in the current experiment. Ivabradine’s HR‐reducing effect improves both supply–demand balance and myocardial energy state, which are considered the dominant factors of ivabradine’s benefit [2]. Moreover, the reduction of LV hypertrophy and collagen content may be of significant value. Cardiac antiremodeling by ivabradine was associated with functional improvement in previous studies in both animals [7, 8, 10] and patients [12]. In the present experiment, ivabradine attenuated SBP decline, which indirectly suggests improved LV systolic function. In addition, the recently observed reduction of serum aldosterone in L‐NAME‐hypertension by ivabradine [4] and the ivabradine’s interference with the renin–angiotensin system in terms of attenuation of the angiotensin II type 1 receptor or angiotensin‐converting enzyme [10] protein expression and diminution of serum angiotensin II [8] may also contribute to cardiovascular protection. Furthermore, the attenuation of the LV structural rebuilding by ivabradine could have alleviated the electrical instability of the damaged myocardium [10] and the occurrence of fatal dysrhythmias. Moreover, besides the bradycardic action, several pleiotropic effects of ivabradine, which seems to be HR‐independent [13, 14], have emerged recently. Anti‐inflammatory and antioxidant actions with improvement of endothelium‐dependent vascular relaxation, collaterals opening, and direct mitochondrial protection might have contributed to ivabradine’s anti‐ischemic action and participate in the cardiovascular protection against isoproterenol‐induced heart damage.

We conclude that ivabradine improved survival in rats with isoproterenol‐damaged hearts.

## Limitation of the study

The limitation of this study was that the attenuation of LV fibrosis and hypertrophy and the alleviation of SBP decline only indirectly reflect a potential improvement of the LV function by ivabradine. Echocardiography could more reliably determine the cardiac function. However, executing echocardiography in general anesthesia (with unavoidable shaving the animal’s chest) could induce an undesirable stress reaction associated with sympathetic overactivation presumably ensuing mortality acceleration in rats with a severe isoproterenol‐induced heart damage, which could distort the results of the survival experiment. On the other hand, performing an echocardiography study in a parallel experiment was beyond the technical possibilities of our laboratory.

## Conflict of interest

The authors declare that there is no conflict of interest.
